# The Hospital. Nursing Mirror

**Published:** 1898-07-16

**Authors:** 


"The Hospital" Nursing Mirror.

Being the Nursing Section of "The Hospital."

[Contributions for this Section of "The Hospital" should be addressed to the Editor, The Hospital, 28 & 29, Southampton Street, Strand, London, W.C., and should have the word "Nursing" plainly written in left-hand top corner of the envelope.]

### News from the Nursing World.

### THE PRINCESS OF WALES AT THE LONDON SCHOOL OF MEDICINE FOR WOMEN.

Very charming did the Princess of Wales look when she visited the London (Royal Free Hospital) School of Medicine for Women on the afternoon of Monday, July 11th. She wore a dress of black muslin with white spots, trimmed with lace, over white; a small hat of heliotrope straw, and a feather boa of the same colour. The room was beautifully decorated, and crammed with people, amongst whom were the Duchess of Portland, Lord and Lady Reay, the Earl of Wemyss, Sir William and Lady Broadbent, Dr. Shuttleworth, Lady Lyall, Mrs. Garrett Anderson, Mrs. Scharleib, Miss Cock, Mrs. Forcett, Miss Aldrich Blake, and others. Madame Antoinette Sterling sang "The Lord is my Shepherd" exquisitely, and without accompaniment. Her notes were bell-like and beautifully modulated. When she finished the Princess turned and thanked her, and there was a general murmur of subdued applause. The procession of young girls and children bearing purses was very pleasing; some of them were so wee that they did not know what to do after placing the purses on the tray, and stood gazing at the Princess with open eyes and mouths until they were gently helped on their way to the other door. The arrangements were excellent, and everything passed off most successfully. The students received many congratulations on the extremely tasteful decorations everywhere. It is not improbable that the Princess will pay a private visit to the schools very shortly.

### THE PRINCESS CHRISTIAN AND THE QUEEN'S NURSES.

It was the privilege of the newly-appointed superintendents and nurses of Queen Victoria's Jubilee Institute for Nurses to receive their badges and certificates from the hands of the Princess Christian at Grosvenor House on Thursday last. This is the town residence of the Duke of Westminster, to whose interest and effort the Institute owes so much of its prosperity. The noble suite of rooms through which the guests passed rank amongst the finest in London, and the richly-painted ceilings, the walls covered with beautiful pictures, the polished inlaid floors and the rare furniture were viewed with much interest. A crimson drugget had been laid down in the room in which the ceremony took place, and the grouping of the blue-garbed nurses on the one hand and the brightly dressed visitors on the other was very effective. The Princess—who looked exceedingly well—and her attendants occupied seats to the right of the small table on which the badges were spread. Her Royal Highness displayed so alert an interest in her task as to make all present feel that it was as great a pleasure for her to bestow the insignia of membership as it was to the nurses to receive them from her.

### THE ANNUAL GATHERING OF THE ROYAL BRITISH NURSES' ASSOCIATION.

After a dull meeting, judged by the standard set by former gatherings of the Royal British Nurses' Association, such members as could spare the time visited the Earl's Court Exhibition, and partook of tea. The weather was delightful, and the gardens presented a gay and animated appearance. There was plenty of music, and hundreds of seats, so that the visitors had every opportunity of enjoying a chat amidst very agreeable surroundings, and of congratulating each other on the prospect of the Association settling down to really good work after the storms and difficulties of the past few years. The Royal President has been much endeared to them by her loyalty to the Association during its trials, and the vote of thanks to her at the meeting brought forth a cordial burst of applause. Next to that of Her Royal Highness, Dr. Bezly Thorne's name was most warmly received, and he could not fail to be touched by the spontaneous manner in which the nurses thanked him for his services on their behalf. The proposal for the original life members to repeat their life subscription of a guinea, and so replace the money expended in securing the charter, was his happy suggestion. He announced his intention of sending in his own contribution in the course of a few days, and several others privately made up their minds to do the same. Mrs. Coster, the newly-appointed nurse hon. secretary, was accorded a hearty welcome, and so, for the first time for some years, the annual meeting of the Royal British Nurses' Association passed off in a highly satisfactory manner.

### THE COLONIAL NURSING ASSOCIATION.

Amongst those present at the second annual meeting of the Colonial Nursing Association, which was held at the Imperial Institute on June 28th, were Lord Loch (who presided), Mrs. Joseph Chamberlain, Lady Musgrave, Lady Quayle-Jones, and Mrs. Francis T. Piggott (hon. secretary). There is undoubtedly a tremendous need of such an association, and the more highly the value of skilled nursing is known, especially in cases of typhoid (so prevalent in our Crown colonies), the more demand there will be for trained nurses, and, let it be hoped, the more liberal will be the support given to the agencies which send them out into the highways and byeways of the Empire. The funds of the society are in a fairly satisfactory state, thanks to the kindness of Sir Squire Bancroft, whose reading of the "Christmas Carol" placed nearly £240 in the exchequer. Nurses are now stationed at Agra, Bangkok, Cyprus, Ceylon, Hong Kong, Lagos, Mauritius, Selangor, and Trinidad. When, however, the necessary "sinews" of progress are provided the work is capable of enormous development.

### THE NURSES' MISSIONARY SOCIETY.

On Thursday morning the Princess Christian opened a loan exhibition and sale of work organised by the Nurses' Missionary Association. The sale took place in the house of the Society for Promoting Christian Knowledge in Northumberland Avenue. The aim of this association is to train students for medical mission work, to make grants to medical missions, and to help to furnish hospitals and dispensaries. For this purpose it strives to interest professional nurses in the cause of those in connection with the Church of England. The monies collected are distributed by the Medical Mission Committee of the S.P.C.K.

### A NURSES' TEA PARTY.

The Hon. Mrs. Drewitt, wife of one of the physicians of the West London Hospital, provided a delightful treat for the nurses attached to it. On Saturday, the 2nd, one-half spent the afternoon in the Botanical Gardens, and on the 9th the second half enjoyed a similar treat. A private omnibus was sent to convey them from and to the hospital, and, as the weather appeared threatening, tea was served under a tent. A drive through the park brought an enjoyable afternoon to a close.

### AN AUXILIARY NURSING SOCIETY.

The Women's Committee on Auxiliaries to the American National Red Cross Relief Committee, of which the Right Rev. Henry C. Potter is the chairman, has organised the "Red Cross Society for Maintenance of Trained Nurses." The Relief Committee expects to provide a hospital ship affording accommodation for 400 sick and wounded. One hundred volunteers, but carefully selected nurses, will be required to staff it, and the expense thus to be incurred is estimated at £5 a month for each nurse. The new "Maintenance Auxiliary" will collect the necessary funds. The right to spend the money on other relief work in the army and navy, if such should prove necessary, is retained by the committee.

### THE ASHTON-ON-MERSEY NURSES' HOME.

Mrs. Coningsby Disraeli, wife of the M.P. for Altrincham, formally opened the Ashton-on-Mersey Nurses' Home on July 8th, the site of which had been given by Sir W. Cunliffe Brookes, who presided. The building fund was partly raised as a memorial of the Diamond Jubilee, and partly by the exertions of Mrs. C. Chetwynd Atkinson. The buildings are intended to accommodate sick and convalescent patients, provision being also made for accidents. The cost is something over £1,100, of which sum only an inconsiderable balance remains to be collected.

### THE GARDEN FETE AT HAWORTH.

It is expected that the Haworth and Oxenhope Victorian District Nurses' Association will gain about £300 by the successful two days' garden fête and sale of work held at Law House, Haworth, the residence of Mrs. George Merral, the president.

### THE JUBILEE MEMORIAL AT NORTHAMPTON.

An excellent site has been obtained on Leicester Parade, Kingsthorpe Road, facing Langham Place, for the new Nursing Institute which is to be Northampton's memorial of the Diamond Jubilee. It has cost £350, and the estimated cost of the institution is £3,000, of which £2,000 is already in hand.

### ROYAL VICTORIA HOME, BRISTOL.

The Victoria Home, Bristol, is the only public or charitable institution established for the reception and treatment of inebriate women in the United Kingdom. Other women guilty of slight criminal offences are also taken charge of. The inmates are treated with kindness, efforts are made to make their lives happy and useful, basket-work and other light industries are taught, and a percentage of the money received is saved for the earner's own use upon her discharge. The inebriates and criminals are kept distinct, the only building used in common being the chapel, but the food for both establishments is prepared in the same kitchens. The establishment has received much favourable notice, and the authorities are anxious for money both to maintain and extend the work.

### THE GUARDIANS' GRANT.

A letter from the Assistant Secretary of the Local Government Board (Mr. W. E. Knollys) to the Guardians of Rotherham Union, bearing upon the perplexing question of Guardians' grants to parish nurses, is of importance. It runs, ". . . with further reference to the proposed contribution by the Guardians of Rotherham Union toward the cost of the maintenance of a parish nurse, I am directed to point out that, whilst this Board would be willing to sanction a subscription if empowered to do so, it appears that there is no 'association or society for providing nurses.' For this reason, the case does not come within the terms of sec. 10 of the 42 and 43 Vic., cap. 54, and I am not aware of any other authority under which payment could be sanctioned." The point which we would emphasise here is that the district nurse must be provided by an organised nursing association before the Guardians can consider the question of making a grant, and that parishes arranging for the services of such a nurse must form a properly constituted association, even if only one nurse is engaged, in order to be eligible to receive help from the poor-law authorities.

### SHORT ITEMS.

A Church Parade of North Yorkshire and South Durham Cyclists was held on June 26th, at Raby, by permission of Lord Barnard. Between three and four hundred persons attended the service conducted by the vicar of Staindrop on the terrace in front of the Castle, the collection being in aid of the Staindrop Nursing Association and the Eden Cottage Hospital, Bishop Auckland. Lord Barnard afterwards entertained the visitors to luncheon in the riding school.—The Cheltenham Board of Guardians has raised the salary of Nurse Hill from £20 to £25 a-year. Her application for the increase was strongly supported by the superintendent nurse.—The Peterborough branch of the Ancient Order of Foresters organised their annual parade on Sunday last in aid of the Peterborough Infirmary, the Peterborough Nursing Association, and the Hunstanton Convalescent Home.—A "rummage" sale, realising the sum of £25 for the Newport and District Benefit Nursing Association, took place recently in the grounds of Mr. Hamilton.—A cheque for £85 has been received by the treasurer of the Barry District Nursing Association, being part proceeds of an assault-at-arms recently held at Cadoxton Theatre.—It appears that the plan of the school nurse set on foot by Miss Honnor Morten is not without precedent. The District Nurses' Association of Hammersmith and Fulham pays regular weekly visits to four of the elementary schools in the neighbourhood, and occasionally to others. Some 1,524 children were attended last year.

### Antiseptics for Nurses.

By a Medical Woman.

### X.—SOME PATENT DISINFECTANTS AND ANTISEPTICS.

There are a very large number of patented disinfectants and antiseptics which have been called after the names of their manufacturers, and consequently there is no clue in the name to the composition of the fluid or powder, whichever it may be. It seems hardly necessary to warn all nurses against being guided entirely by the manufacturers' report of the value and uses of his compound, without endeavouring to find out the component parts and considering whether the disinfectant properties of these are trustworthy or not. Of course, it is always possible, and perhaps desirable, for the cautious person to keep to the recognised antiseptics and limit herself to the use of carbolic acid and perchloride of mercury whenever the choice of the antiseptic or disinfectant rests exclusively with her. On the other hand, however, such a restriction if universally practised would entirely preclude the use of many of the phenol preparations which have already proved so invaluable in surgery.

To enable nurses to judge of the value of the various patent antiseptics and disinfectants with which they may meet, and as to the advisability of using which they may be consulted by their patient, we now propose to give the components of some of those patents with which they are likely to be confronted during the discharge of their vocation.

Tuson's Disinfectant consists of a strong solution of chloride of zinc impregnated to saturation with sulphurous acid gas. The value of this solution as a disinfectant depends on the fact that the sulphurous acid gas is readily liberated from the liquid when the latter is exposed in vessels to the air. Hence it is an easier way of obtaining sulphurous acid gas when such is required than by the older method of burning sulphur. The fluid is always coloured green so as to avoid accidents.

Tuson's Disinfecting Powder is another preparation by which sulphurous acid gas is easily procurable. It is a mixture of calcium sulphite and aluminium and zinc sulphates, prepared in the form of a fine powder of a light green colour. It is only necessary to moisten it with water when the gas is evolved.

Lawes' Disinfectant Fluid is derived from one of the phenol preparations which, being insoluble in water, is kept in solution by means of soda. It is a brown fluid with a pleasant odour like tar, but when mixed with water it forms a milky emulsion. It forms an efficient antiseptic in the proportion of 1 part of the fluid to 100 of water.

Esset's Fluid is in many respects similar to Jeyes' Fluid, being a tar preparation, and occupies much the same position as a disinfectant and purifier. It is a fluid, of a dark brown colour, and when mixed with water, which it does very readily, it forms a white milky emulsion. It is a good and efficient antiseptic, but we do not think it is "the strongest known," nor can it be correctly described as "non-poisonous." It should be used as a 1 per cent. solution for drains, &c.

Burnett's Disinfecting Fluid. In this patent the active disinfectant is chloride of zinc of the strength of 25 grains of zinc chloride to each drachm of the fluid. It is an excellent disinfectant for drains if used in the proportion of a pint to a gallon of water, but is poisonous, and is injurious to living tissues.

St. Bede's Disinfecting Fluid is composed of perchloride of mercury and sodium sulphate as active disinfectants, but with the addition of eucalyptus, thymol, and sulphuric acid, and consequently it is very efficient. It can be obtained either as a fluid or in small blue blocks, the colour being added to distinguish the fluid from plain water. Each block gives a certain quantity of liquid disinfectant of known strength.

Andeer's Antiseptic Lotion: In this the active antiseptic substance is resorcin, which is derived by various processes from carbolic acid, and hence is allied to that antiseptic. Resorcin in a dry state consists of white lustrous crystal-like needles, white when pure, but, like pure carbolic crystals, turning pink when exposed to the air. It is soluble in water in the proportion of 1 part resorcin in 2 of water, and forms a solution having a faint odour like carbolic, and with a sweet and slightly pungent taste. Resorcin is a good antiseptic without being an irritant, and should be used for that purpose in a solution of the strength of 2 to 10 per cent.

Bond's Antiseptic Pellets contain perchloride of mercury and sodium chloride or ordinary salt, the latter being added to prevent the solution from turning milky when made with ordinary hard tap water. One pellet in a pint of water makes a solution of perchloride of mercury of 1 in 2,000 strength.

Wilson's Antiseptic Solution is another form of perchloride of mercury to which an equal proportion of sal ammoniac has been added, the use of ammonia and of salt being to prevent the perchloride of mercury from forming an insoluble albuminate with the exudation from the wound, and so interfering with the action of the antiseptic and rendering it useless.

Salufer: In this the active antiseptic principle is fluorine, a gas obtained by heating fluor-spar with strong sulphuric acid. Salufer, however, as prepared from various fluorides is a powder, without odour and not very soluble in water. It is sold in the form of convenient sized blocks from which a solution of known strength can be made. It is said to be a powerful but non-irritating antiseptic.

Loretin: Here the active principle is one of the coal tar derivatives. It occurs as a powder having an agreeable odour, and is a good non-irritating antiseptic, which can be applied to wounds in place of iodoform.

Payen's Patent Disinfectant is a very irritating gas prepared by pouring nitric acid on copper turnings, used for disinfecting rooms. It has, however, no advantages over sulphurous acid, which is obtained by burning ordinary sulphur, and is more difficult to produce.

Microcidine contains the two antiseptics naphthol and phenol, and hence is allied to the coal tar products. It is not a very satisfactory antiseptic owing to its irritant properties.

Radlauers's Antisepsin consists of sulphate and iodide of zinc, thymol, and boracic acid, and is a fair but somewhat irritating antiseptic.

Other patent antiseptics in which the active principle is zinc are the following: Raymond's Antiseptic, Disinfecting, and Deodorising fluid, which also contains a small quantity of iodide of mercury; Rotterine, in which zinc is combined with other disinfectants, such as boric acid, salicylic acid, and thymol; Eau Larnaudes, which also contains some copper sulphate; Eau de Saint Luc is extensively used as a disinfectant in France. Two other common French disinfectants are Eau de Javelle and Eau de Labarraque; the former is chlorinated potash, and the latter chlorinated soda.

### Presentations.

Miss Mottram, Superintendent of Nurses' Glasgow Royal Infirmary, who has been appointed Lady Superintendent of the Kent Nursing Institution at Tunbridge Wells, was presented on leaving Glasgow with a handsome silver (Queen Anne) tea set by the nurses. She also received several presents from individual friends in appreciation of her work during the last eight and a half years. Miss Mottram is a member of the Royal British Nurses' Association and a registered nurse.

### Royal British Nurses' Association

THE annual meeting of the Royal British Nurses' Association, which was held in the East Conference Hall of the Imperial Institute on Tuesday, the 12th inst., was characterised by a unanimity which has been absent from the meetings of this Association for a very long time. Hardly a discordant note was sounded throughout the meeting, and with one exception the voice of the opposition which has for so long made these meetings a series of unpleasant personalities, was not heard. Sir JAMES CRICHTON BROWNE occupied the chair, and was supported on the platform by Mr. Pickering Pick, Dr. Bezly Thorne, Mr. Alfred Cooper, Mr. John Langton, Mr. E. A. Fardon, Miss Thorold, and Mrs. Coster.

FINANCIAL REPORT.

The minutes of several special meetings having been confirmed,

The TREASURER made his financial report for the year ended March 31st, 1898. The accounts which he presented showed that the receipts were as follows: General account (including a balance of £144 brought forward), £1,021; journal account, £140; registration account, £125; total, £1,286. The expenditure amounted to £1,062, made up as follows: General account, £741; journal account, £170; registration account, £151; leaving a balance at the bank of £224. Thanks to Miss Foggo Thomson and Miss Danks the Benevolent Fund had been enriched by a sum of £107. Mr. Langton moved the adoption of his report and the accounts.

Mr. ALFRED COOPER, in seconding, said that he was glad to see that the treasurer had succeeded in bringing the normal expenditure of the Association within the limits of its income.

Miss DE PLEDGE supported the motion, and spoke very highly of the devotion and ability the Treasurer had shown in carrying on the affairs of the Association. (Loud applause.) She trusted that the Association was now fairly launched and that a prosperous future was before it. It had had a very hard struggle in the past, but now she hoped the members would no longer have occasion to be ashamed of their Association. (Applause.)

Dr. BEDFORD FENWICK said that the Treasurer had omitted, quite inadvertently he was sure, to mention one or two points. The first was the very significant certificate of the auditor in which he stated that "the liabilities are stated to amount to about £250, including £68 for rent, £65 for printing the register, and £100 loan from the hon. treasurer." As the balance at the bank amounted to only £224, the fact remained that at the end of the year the association was considerably in debt. Then the small number of annual subscribers was very depressing to those who had the interests of the Association at heart. Roughly only 1,290 members had paid their subscriptions during the past year, and as the life subscriptions had been spent this was a very serious state of affairs. He was informed that in consequence of the new bye-laws a very large number of the leading members of the association were resigning, and that thirty of the leading hospital matrons of the country had sent in their resignation and others were doing the same thing. Seeing that these ladies had taken a most active part in founding the association, and that under their authority there were many hundreds of the best-trained nurses in the country, their secession would be a most deplorable blow to the Association. He cordially endorsed all that anyone could say with regard to the value of the assistance Mr. Langton had rendered to the Association.

The TREASURER, replying to Dr. Bedford Fenwick, said that the observations he had made were just the same as those he (the Treasurer) had already replied to at the last annual meeting. As to the question of the life subscriptions which Dr. Bedford Fenwick had raised, he would like to point out this: The investments in 1891 amounted to £1,340, in 1892 they were reduced to £1,060, the next year they had disappeared. He did not say that the money was not rightly spent, but it was spent to a great extent in procuring the charter. These investments were the outcome of the life subscriptions, and he held somewhat strongly that these investments ought never to have been touched. (Applause.) They carried obligations for many years, and if people wanted a charter they should have put their hands into their pockets to pay the expenses. (Applause.) In conclusion, he urged each nurse member to work for the Association as if the whole matter depended upon her own efforts alone. (Loud applause.)

The adoption of the report was carried.

ELECTION OF GENERAL COUNCIL.

The CHAIRMAN said the report of the scrutineers appointed to examine the ballot papers sent in by the members for the election of the new General Council showed that the Council as proposed by the Executive Committee had been elected by a large majority. The scrutineers reported that 694 papers had been returned signed and unaltered, 28 signed and altered, and 15 unsigned.

Miss DE PLEDGE formally moved that the Council as proposed by the Executive Committee be elected.

This was seconded and carried.

REPORT OF THE EXECUTIVE COMMITTEE.

Mr. E. A. FARDON presented the report of the Executive Committee. In moving the adoption of the report, he bore witness to the debt of gratitude the Association owed to H.R.H. the Princess Christian, their president. Throughout all the serious time that the Association had passed through this illustrious lady had never once flinched from her determination to see the Association flourishing, and there was no doubt that it had been to a great extent due to the continuance of her gracious countenance that the Association stood where it now did. He then dealt with the effect which the new bye-laws would have upon the Association, saying that under these bye-laws, for the first time in its history since the granting of the charter, the Association would be a free and independent body.

Mr. FRANCIS HAWKINS seconded, and the report was agreed to.

VOTE OF THANKS.

Mr. T. PICKERING PICK proposed, and Miss WEDGWOOD seconded, a vote of thanks to the President, which was carried unanimously.

Dr. BEZLY THORNE, who was greeted with loud applause, proposed a vote of thanks to the honorary officers, and in the course of his speech suggested that all those life subscribers whose subscriptions had been absorbed in the way the treasurer had mentioned, should pay up their subscriptions again. He did not think many of them would mind doing that, and he for one would do so. (Applause.)

Miss SCOTT seconded, and the vote of thanks was agreed to.

The CHAIRMAN, speaking on behalf of the honorary officers, said that they all realised what a great debt of gratitude they owed to Mr. Fardon, who had bravely borne a most onerous burden for the past three years. (Applause.)

Mr. FARDON replied, and the proceedings shortly afterwards terminated.

### Where to Go.

A GRAND morning concert will be given at Lady McKenna's, 67, Lancaster Gate, on Thursday, July 14th, at three p.m., in aid of the Royal Society for the Prevention of Cruelty to Animals.

### Training in the Provinces.

(*Continued from page* 126.)

### BRISTOL GENERAL HOSPITAL (200 Beds).

### Terms of Training.

Probationers are received for training at the Bristol General Hospital between the ages of 23 and 35. A months' trial is required of candidates, and the term of training is for one or three years. An entrance fee of £25 is asked from those who wish to have one year's training only. No salary is given for the first year, payment beginning at £14 the second year, increasing to £16 during the third. Laundry expenses are defrayed by the hospital; and uniform, consisting of print dresses, aprons, and caps, is given to each nurse at the expiration of her month's probation, and renewed yearly. Nurses are expected to provide outdoor uniform for themselves. If a nurse leaves the hospital before the end of the first year the estimated value of the uniform given to her by the hospital is deducted from her salary.

Certificates are only granted to nurses who have satisfactorily completed the full three years' course of training. Lectures are given by members of the medical staff, and practical instruction in nursing by the matron, prizes being offered at the yearly examinations.

At the completion of the three years' training those nurses who are suitable may be promoted to appointments on the private nursing staff, and afterwards to posts as sisters of wards. Salaries paid to nurses on the private staff begin at £26 per annum, rising to £30. Sisters receive £35 a year.

Night duty is undertaken alternately with day duty by the second and third year nurses.

### Hours On and Off Duty.

The working day is from 7.30 a.m. to 8 p.m., sisters going on duty rather later in the mornings. Half an hour each is allowed for lunch, dinner, and tea during these hours, and between two and three hours' leave for recreation each day. Nurses go off duty on alternate mornings between 9 and 12 o'clock, and on alternate afternoons from 2.15 to 4.30. On alternate Sunday evenings leave of absence is granted from 4 to 9, and one whole day each month. Staff nurses have two afternoons and two evenings free during the month, besides the "day off," and sisters are free every alternate evening, and once a month from Saturday till Monday. Three weeks' annual holiday is allowed to probationers, and one month to sisters.

### Meals.

The hours for meals are as follows: Breakfast, 7 a.m. (sisters, 7.45); lunch, 10 to 10.30 a.m.; dinner, 1 p.m. (sisters, 12.30); tea, 4.30; supper, 9 p.m. All meals for the nurses, including lunch and tea, are served in the dining-room, the latter meals for the sisters being sent to their own rooms. Allowances of sugar, butter, &c, are only given out to the night nurses for their midnight meal.

### BRISTOL ROYAL INFIRMARY (270 Beds).

### Terms of Training.

All probationers are received at the Bristol Royal Infirmary upon identical terms, *i.e.*, for three years' training at a salary of £8 the first year, £16 the second, and £18 the third. Candidates should be between 23 and 30 years of age. The certificate is given upon satisfactorily completing the three years' engagement and passing an examination, lectures being given by members of the medical staff upon medical and surgical nursing. In consequence of a maternity department being attached to the hospitals probationers are enabled, if they so wish, to qualify for the L.O.S. certificate. Indoor uniform is provided by the hospital, and laundry expenses also. All probationers are alike eligible for promotion to the post of ward sister, or to the private nursing staff attached to the hospital, according to qualifications and capability. Sisters' salaries range from £30 to £35, and on the private staff the salaries are from £26 to £30 per annum. Night duty is taken by second and third year nurses in alternation with day duty.

### Hours On and Off Duty.

Probationers go on duty in the wards at 7.30 a.m., and leave them in the evening at 9.30. They are daily off duty from 2 to 3.30 p.m., with time for meals, and have half a day once a month. Assistant nurses' hours are nearly the same except that instead of two hours' daily recreation they are off duty on alternate days from 4 to 8 p.m., and they have leave of absence on alternate Sundays from 4 to 9.30 p.m. Sisters' hours on duty are from 8 a.m. to 10 p.m., with time for meals, being off duty on alternate days from 4 to 8 p.m.; on alternate Sundays from 4 to 9.30 p.m.; with a whole Sunday and a half day once a month. Night nurses are on duty from 9.30 p.m. to 9.30 a.m.; off duty daily from 10 a.m. to 12.30 p.m., on Sundays from 9.30 a.m. to 1 p.m. or 5 to 9 p.m., and they have a night off once a month. Yearly holidays are for sisters, night, and assistant nurses, three weeks; for probationers, two weeks.

### Meals.

Probationers and assistant nurses' hours for meals are as follows: Breakfast, 7 a.m.; dinner, 12 noon; tea, 3.30 p.m.; supper, 8.30 p.m. Sisters' breakfast at 7.30 a.m., their dinner is at 11.30 a.m., and supper at 8 p.m. All the nurses' meals are served in the dining-rooms. The sisters' tea is served in what are called the "side rooms," and allowances of tea, sugar, and butter, &c., are given out to them for this purpose. The allowances for the nurses are given in charge of the dining-hall maid.

### SUSSEX COUNTY HOSPITAL, BRIGHTON (190 Beds).

Candidates for training at the Sussex County Hospital should be between 23 and 30 years of age. The term of training is three years, for which a premium of £35 is required, no salary being given until the full course is completed. Lectures on medical and surgical nursing, anatomy, and physiology, &c., are given by the matron and by the members of the medical staff, and certificates are given at the end of the three years. Nurses are required to provide their own uniform, but laundry expenses are defrayed by the hospital. Pensions are granted to nurses after 20 years' service.

For promotion to the post of sister of wards a year's training elsewhere is usually considered desirable, but sisters are sometimes selected from those probationers who have satisfactorily completed their three years' training.

The night nursing is done by probationers in their second and third years, taking three months alternately with day duty.

Staff nurses receive a salary of £25 per annum, sisters from £37 10s. to £42 10s., this being taken to include cost of uniform. There is a private nursing staff, which nurses may join on completing their training in the wards, at a salary of £40 a year, with share in the profits.

### Hours On and Off Duty.

Every sister and nurse has not less than two hours off duty daily with time for meals. The working day is from 7 a.m. to 8.45 p.m., including the off duty and meal times. A whole day off is given once a month, on which the nurses do not make their appearance in the wards at all. A month's holiday is allowed during the year.

### Meals.

All meals are served in the dining-rooms. Sisters have certain weekly allowances, but none are given into the charge of the nurses themselves. Hours for meals are as follows for the day staff: Breakfast, 6.30 a.m.; lunch, 10 a.m; dinner, 1 and 2 p.m.; tea, 5 p.m.; and supper, 8.30 p.m.

### The Queen's Nurses at Grosvenor House.

H.R.H. PRINCESS CHRISTIAN OF SCHLESWIG-HOLSTEIN, accompanied by Lady Emily Lock and Major Evan Martin, visited Grosvenor House on Thursday, July 7th, at half-past three p.m., and bestowed the badges and certificates on Queen Victoria's Jubilee Nurses. The Rev. A. L. B. Peile, master of St. Katherine's and president of the Queen Victoria's Jubilee Institute for Nurses, took the chair, and the Duke and Duchess of Westminster were present, receiving the guests who came to witness the pretty ceremony. Amongst them were the Hon. Sydney Holland, Sir Dyce Duckworth, M.D., Mr. Henry Bonham-Carter, Mr. Harold Boulton, Lady Lucy Hicks Beach, Lady Victoria Lambton, Mrs. Theodore Acland, Mrs. Cheadle, and Miss Rosalind Paget, all members of the Council. The nurses whose names are added at the end of this notice occupied the chairs arranged on one side of the great drawing-room, the superintendents wearing the dark blue dresses and bonnets, the nurses the blue print dresses, bonnets, and white aprons, the uniforms of the institute; the guests were seated on the other side of the room. The Princess, who was dressed in a navy blue china silk with a white pattern, and wore a black toque trimmed with damask carnations, entered from the garden. The Duke of Westminster in a few words related some points in the rapid progress of the Institute. The nurses might be proud, he said, of belonging to the great and thoroughly trained army engaged in soothing and alleviating the suffering caused by disease to Her Majesty's poor subjects in England, Scotland, Wales, and Ireland. Last year had been a memorable one in the annals of their Institute; the Queen herself had received her nurses at Windsor, and a large sum of money had been raised to extend the work. His Grace concluded by asking the nurses not to hurry away unless obliged to do so, as music in the garden and light refreshments had been provided for their entertainment.

The Princess Christian presented the silver badges to the superintendents first and then to the nurses, the chairman calling the names, and Miss A. Martin Leake, the secretary, handing her Royal Highness the badges and certificates. Each nurse advanced as her name was pronounced and received her award.

At the end of this pleasing ceremony the master of St. Katherine's delivered an appropriate little homily, recalling the responsibilities of their position to the superintendents and nurses. Sir Dyke Duckworth followed, endorsing the president's remarks and urging upon those wearing the badge of the institution to remember their high mission—they were carrying succour in the Queen's name to the sick and sorrowful, instructing the world in the value of trained nursing, and refuting the fallacious arguments of those who believed that willing hearts made amends for unskilled hands, and in consequence advocated the introduction of an inferior grade of nurses into the cottages of the poor.

The Princess then withdrew to the garden. The nurses and other guests found tea, coffee, cakes, and fruit prepared in the dining-room on a long table, made beautiful with silver and splendid Malmaison carnations.

The number of nursing associations affiliated to the Queen Victoria's Jubilee Institute for Nurses, July 1st, 1898, were: England, 206, and five county associations; Scotland, 100, and one county association; Ireland, 43; Wales, 50; total, 399, and six county associations. The Number of "Queen's" nurses employed on July 1st, 1898, was 760.

The number of nurses enrolled as "Queen's" Nurses in 1898 was as follows:—

| | Jan. 1st. | July 1st. | Total. |
|---|---|---|---|
| England | 54 | 65 | 119 |
| Scotland | 16 | 21 | 37 |
| Ireland | 10 | 8 | 18 |
| Wales | 8 | 9 | 17 |
| | 88 | 103 | 191 |

The following are the names of nurses receiving badges at Grosvenor House, July 7th, 1898:—

I. For silver badges.—Present: Mary Armstrong, assistant inspector; Caroline Anne Blackmore, assistant superintendent, Irish Branch; Isabella Cairnie, superintendent East London Nursing Society; Elizabeth Harriet Curtis, superintendent Hammersmith and Fulham District Nursing Association; Annie Julia Franklin, superintendent Chelsea and Pimlico District Nursing Association; Annie Michie, superintendent Cornwall County Nursing Association; Florence Saunders, superintendent Peterborough District Nursing Association; Georgina Mabel Shalders, superintendent St. Olave's District Nursing Association; Arabella Elizabeth Stone, superintendent Westminster Nursing Committee; Elizabeth Waller, superintendent Birmingham District Nursing Society; Laura Lottie Ward, superintendent Southwark, Newington, and Walworth District Nursing Association. Absent: Wilhelmina Edith Dodds, superintendent Camberwell District Nursing Association; Mary Jane Murphy, superintendent Southampton Branch of Institute.

II. For certificates on the completion of two years' service: Hilda Goadby, working at Tunbridge Wells; Jessie Coleman, working at Alfreton; Eleanor Purton, working at Camberwell; Emily Jane Tipping, working at Rotherhithe; Clara Uridge, working at Bognor.

III. The names of those receiving bronze badges on appointment as "Queen's" nurses appeared in the "Mirror" of July 2nd, 1898.

### Appointments.

### MATRONS.

KAISR-EL-AINI, CAIRO.—Miss Caroline H. Glover, matron of the Manchester Royal Eye Hospital, has been appointed Matron of the Kaisr-el-Aini Hospital under the Egyptian Government. Miss Glover was trained at the Nightingale School, and was afterwards staff nurse and sister of the ophthalmic ward of St. Thomas's Hospital. She then went to the Hospital for Sick Children, Great Ormond Street, where she was staff nurse and later home sister. She became matron of the Kent and Canterbury Hospital in December, 1894, and was appointed matron of the New Sanatorium, Shrewsbury Schools, the following January.

SWANSEA HOSPITAL.—Miss A. E. Sherring was elected Matron of this hospital on July 4th. She was trained at the Liverpool Southern Hospital, and her subsequent appointments have been as follows: Charge nurse at Southport Infirmary, charge nurse at the South-Eastern Fever Hospital, London, S.E., sister at the Stanley Hospital, Liverpool, and night superintendent of the Swansea General and Eye Hospital, which post she resigns to assume her new duties.

PERSHORE COTTAGE HOSPITAL.—After two and a half years' service Miss Jesse has resigned her position as matron, and has accepted an appointment at the Queen's Hospital Nursing Institution at Birmingham. Miss Fanny Gertrude Digwood, who was trained at and afterwards on the private staff of the Warneford Hospital, Leamington, has been appointed Matron.

INGHAM INFIRMARY, SOUTH SHIELDS.—Miss H. L. Mackridge was selected out of fifty applicants Matron of this infirmary on July 5th, 1898. She was trained and successively held various posts at the General Infirmary, Leeds, where she is now Sister-in-charge of the surgical and accident ward.

A CORRECTION.—Miss Ellen E. Wilson's appointment as Superintendent Nurse of Romford Union Infirmary was unfortunately misplaced last week. Miss Wilson, in addition to her other qualifications, holds the certificate of the L.O.S.

### Minor Appointments.

ST. MARK'S HOSPITAL FOR FISTULA AND DISEASES OF THE RECTUM, CITY ROAD, E.C.—Miss May Trenory, who received two years' training at the Colonial Hospital, Gibraltar, and three and a half years at the London Hospital, was, on July 12th, appointed Night Sister of the above hospital.

NEWPORT AND MONMOUTHSHIRE HOSPITAL.—Miss H. Saule, who was trained at Cardiff Infirmary, has been appointed Charge Nurse of this hospital.

### London School of Medicine for Women.

(ROYAL FREE HOSPITAL.)

### OPENING OF THE PFEIFFER WING.

ON Monday, July 11th, the Princess of Wales, accompanied by the Prince of Wales, Miss Knollys, and Colonel Clarke, visited the London (Royal Free Hospital) School of Medicine for Women to open the new science laboratories. These are known as the Pfeiffer Wing, as the expense incurred in building it was partly defrayed by the Pfeiffer bequest, partly by the surplus of income over expenditure, and partly by public subscription, which includes a donation of £1,000 from Miss Payne Townshend. It consists of four large class rooms for physics, chemistry, physiology, and anatomy, one over the other, each resembling greatly the wards of a hospital. There are numerous windows opposite each other down the sides and the end is rounded. The outside effect is very ordinary, the wing being simply a solid oblong block of red brick with plain unbroken lines, relieved somewhat by the massive roof and the supports curving outward to meet it. Inside it is spacious, admirably adapted to the purpose intended, and capitally ventilated. The fittings are very complete, especially in the physiology class-room. It was worth while to visit the old class-rooms to note the contrast. There the ricketty attics, rudely pierced with skylights to admit the necessary light, the floors covered with sheets of zinc, the necessary appliances of the humblest description were mean, dilapidated, and covered with dust—a silent but most impressive witness of the need of the new building. The opening ceremony took place in the class-room on the ground floor of the new wing. A gangway had been erected between it and the students' recreation room in the old building, where tea was served to the Princess and her suite. Her Royal Highness arrived at four o'clock, and was welcomed by Mrs. Garrett Anderson, M.D., in a short speech giving a digest of the history of the movement for the medical education of women and stating the number of former pupils now earning an honourable maintenance throughout the world. The Bishop of London (Dr. Mandell Creighton) said the Lord's Prayer and three collects suitable for the occasion from the Liturgy, after which Madame Antoinette Sterling sang most expressively "The Lord is My Shepherd," at the conclusion of which the Princess declared the science laboratories open, and the Prince of Wales then addressed those assembled, expressing the pleasure it gave them both to be present on account of the deep interest they took in the progress of the higher education of women, and the delight they experienced in hearing of the success of former students in different parts of the world, especially in the Zenanas of India, where, from religious scruples, the male practitioners were excluded. He said that two days before he had paid a private visit to the school, and was much impressed by the assiduity with which the pupils pursued their studies; he had also recently visited the New Hospital for Women, of which the Princess of Wales laid the foundation-stone some years ago, and the whole of the staff of which consisted of ladies who, he believed, had become graduates by working at this school. After this a number of children entered in single file and laid purses on a tray in front of her Royal Highness. The contents represented sums of from £5 upward. Altogether, since the Princess announced her intention of opening the new laboratories, about £4,000 have been received in contributions towards the building of a second wing. The loss of the students' tennis lawn—necessitated by the recent extension—has been fairly well remedied by acquiring other tennis courts in the Botanical Gardens.

### Everybody's Opinion.

[Correspondence on all subjects is invited, but we cannot in any way be responsible for the opinions expressed by our correspondents. No communication can be entertained if the name and address of the correspondent is not given, as a guarantee of good faith but not necessarily for publication, or unless one side of the paper only is written on.]

### TRAINING IN THE PROVINCES.

The matron of the Birmingham and Midland Eye Hospital, writing in reference to the notice of that institution under the above heading in a recent number of THE HOSPITAL, asks us to mention that the nurses have an airy dining-room at the top of the building where meals other than lunch and tea are served. We should also add that in consequence of a clerical error the periods of night duty taken by the nurses were given as six months instead of six weeks.

### COLLECTING A NURSE'S FEES.

"VICTORIA" writes: I shall be much obliged if you can advise me in the following matter. What can a private nurse do who is working on her own account in the matter of fees. I, belonging to an institution, hand in a paper to my employer with rules which ask in long-standing cases of illness for fees to be paid monthly, but a friend of mine who is working on her own account has sometimes difficulty in getting paid her full fee or at the right time. I should be very glad if some private nurse working alone would give advice as to whether she has any printed form to give her employer which would do away with any discussion at the end of the case as to whether the fee is or is not a fair one.

[A private nurse should certainly hand her employers a definite scale of charges, or make her agreement with them in writing. Unless satisfactory reasons are given for failing to pay the amounts due punctually the nurse should insist upon an immediate settlement, as nothing is to be gained by letting the weekly or monthly payments accumulate. The enunciation of the principle is easy enough, but there are so many perplexing issues involved in practising it that the experience of others will certainly be helpful.—ED. *T.H.*]

### THE MATRONS' COUNCIL.

"OLD RE——L" writes: In reply to "Sister Mary," I am sure I can say a word for my training school, even though it is a provincial infirmary. There was a staff of thirty-two nurses and one resident medical officer. So who did the work? for there were between four and five hundred beds, very often all occupied. All dressings were left to the charge nurses and pros. (under their direction). We had lectures from the house surgeon and the visiting physician. We had also for one hour every Saturday evening from the Superintendent bandaging classes, as well as extra classes occasionally for invalid cookery. Then there were splints to pad during the week, and no excuse would be taken for missing these. A friend of mine when a "pro." in a training school which considers itself head and shoulders above mine because of the city it is in, once said to me, "I can come out and meet you all right, it's only bandaging class, and I havn't been at it for weeks." When we were examined for our certificates—I should have said before we had exams. after each course of lectures—an outside doctor was the examiner, and it took this form: Thirty-six questions were asked of each nurse separately, and answered by mouth; sixteen questions were written, three hours being allowed for answering. Then the examiner, with the visiting physician, house surgeon, and lady superintendent adjourned to a ward set apart for the purpose that day, and we had to make beds and poultices, bandage the doctors, rig up tent beds, and from a table bring any instrument which they chose to ask for by its full proper name. We were also taught how to test urine for albumin, sugar, pus, blood, and bile pigment. We had also a few classes for dispensing, but I think the utmost we managed to do with that was to upset the surgery. There are lots of nurses who will recognise this letter and say "I am certain that is from 'Old Re——l.'"

### For Reading to the Sick.

"OUR WISE AND LOVING KING."

**Verses.**

It remains, if thou, the image of God,
Wilt reason well, that thou shalt know His ways.
But first thou must be loyal! Love, O man,
Thy Father; hearken when He pleads with thee,
For there is something left of Him e'en now,
A witness for thy Father in His soul,
Albeit thy better state thou hast foregone.
—*J. Ingelow.*

For the love of God is broader
Than the measure of man's mind,
And the heart of the Eternal
Is most wonderfully kind.
But we make His love too narrow
By false limits of our own,
And we magnify His strictness
With a zeal He will not own. —*Faber.*

The heights by great men reached and kept
Were not attained by sudden flight;
But they, while their companions slept,
Were toiling upward in the night;

Standing on what too long we bore
With shoulders bent and downcast eyes,
We may discern—unseen before—
A path to higher destinies.

Nor deem the irrevocable past
As wholly wasted, wholly vain,
If, rising on its wrecks, at last
To something nobler we attain. —*Longfellow.*

**Reading.**

God is our King. We are his subjects. A humble life means a life that is subject to Him. Of course, if we are His subjects, there cannot be any hardship in being "subject to God." It is only what is right and fit. What happens so very often, however, is this—that our King tells us He wishes us to do one thing, and our own wilfulness tries to make us do something else.

This is God's will and man's will crossing one another. God's will always points up to Heaven, and man's will, man's own self-will, goes along level with the earth; together they form a "cross," and it was because man's will was contrary to God's will that the Son of God died upon the Cross. He has given us everything we possess, every power of soul or mind or body, and we have to choose what we will do with these good gifts. Shall we use them against God, as the soldiers did who used the muscles that God had given them to drive the nails through His hands, or shall we bring them all into subjection, offering to Him a willing service, giving up ourselves, our souls and bodies, that we may be His voluntary subjects? . . . And it is by submitting ourselves to Him in little things that we shall learn to become His faithful subjects. . . . This is often hard to do, even though the matter is a small one, but fortunately for us, the grace to do it may be had for the asking, and every time we bend our will to His we have taken a real step in making ourselves subject to Jesus.

After we have done this we always find that His way is really the best, that it has a pleasure peculiarly its own, and that "His ways are ways of pleasantness and all His paths are peace."—*H. W.*

### Wants and Workers.

NURSE HARDING, Nightingale Home, Osborne Road, Southsea, wishes to buy a second hand copy of Quain's Medical Dictionary.

### Notes and Queries.

**The contents of the Editor's Letter-box have now reached such unwieldy proportions that it has become necessary to establish a hard and fast rule regarding Answers to Correspondents. In future, all questions requiring replies will continue to be answered in this column without any fee. If an answer is required by letter, a fee of half-a-crown must be enclosed with the note containing the enquiry. We are always pleased to help our numerous correspondents to the fullest extent, and we can trust them to sympathise in the overwhelming amount of writing which makes the new rules a necessity. Every communication must be accompanied by the writer's name and address, otherwise it will receive no attention.**

*The Termination of an Engagement.*

(146) Is the matron right in the following instance: At the termination of a month's notice, a nurse made arrangements to leave the hospital in the afternoon of the day the engagement ended. The matron, hearing by chance the evening previous to her leaving that she had done so, claimed her services up to the night of the day of her departure?—*Nurse.*

If a nurse is paid up to and including the day in which her notice expires, the matron might be within her rights in requiring her services till the day's work is over. In many institutions, however, there is an understanding that a nurse may go off duty at noon on the day she leaves, and it never can be proper for women to be made to leave their situations in the evening. It would have been more courteous if the nurse in question, before making her arrangements, had consulted the matron's convenience, who no doubt would have met her wishes in the matter.

*Wanted, a Bicycle.*

(147) I desire to buy a second-hand bicycle. I am doing daily work, and a bicycle would be a great help to me.—*Florence C.*

We cannot become agents for the sale of bicycles. Why not write to the "Exchange and Mart," or to a cycling journal?

*Housekeeping.*

(148) Can you tell me where I can obtain lessons in housekeeping to qualify for a post either in an institution or a private family?—*Housekeeper.*

You can get lessons in the various arts necessary to housekeeping, *i.e.*, book-keeping, cooking, and such like, at a polytechnic institution. To qualify yourself for work in a private family or an institution, you must go as assistant housekeeper or matron.

*Wanted, a Home.*

(149) Having been left a widow with two children, I should like to get into a home for such, where I could help, and have my children with me. I have had three months in an infirmary, and can give excellent testimonials. Can you tell me of one?—*Meg.*

You ask a difficult thing, and give no particulars as to your own or your children's ages. Neither do you say if they are boys or girls, nor what qualifications you possess except the three months in an infirmary. Under these circumstances, it is impossible to advise you. We can only suggest that you should try and get a post as matron in a school where your children could become pupils.

*Training.*

(150) Can you tell me of a cottage or a children's hospital where I could be received as a probationer, receiving salary and uniform. I have been a domestic servant, and am 23 years old. I have applied at several of the hospitals mentioned in Honnor Morten's book, but have been unsuccessful so far.—*A Reader, Gloucestershire.*

As most matrons require a personal interview before engaging a probationer, your living in the country is a drawback. Could you not call (by appointment) on the matron of your nearest training school, and ask her advice? You are of the right age, and, if suitable in other respects, there are plenty of hospitals that would train you, especially under the poor law. There are advertisements for probationers in our columns every week. See St. Saviour's Union Infirmary, p. xi. in "Mirror" for July 9th. The matron, Victoria Hospital, Blackpool, advertises in the same number.

*Probationer of 40.*

(151) Kindly inform "Long Desire" if there is any hospital or institution where she can acquire practical knowledge of nursing. She is 40 years of age, and is quite willing to pay a small sum weekly to obtain such knowledge.

See our rules to correspondents at head of column.

*An English Translation.*

(152) Is there an English translation of Professor Schenk's medical book lately published in Vienna?—*B. E. C., Herts.*

"The Determination of Sex," by Dr. Leopold Schenk, is published by the Werner Company.

*Outfit for Alexandria.*

(153) Could you tell me what outfit a nurse would require at Alexandria for two or three years? 2. Is there a handbook on poisons and their antidotes for nurses?—*Nurse Alice.*

1. You will require very much the same outfit as for an English summer, with the addition of washing coats and skirts and light walking shoes. If nursing, you must have warm dressing-gown and slippers, for the nights are bitterly cold. 2. "What to do in Cases of Poisoning," by Wm. Murrell, M.D., F.R.C.P., price 3s. 6d. net, from the Scientific Press, London.

*Insurance against Sickness.*

(154) Can you inform me if there is an insurance office in London where ladies can insure against sickness as well as accidents?—*F. C. L.*

The Royal National Pension Fund for Nurses gives its policy-holders this option. Apply to the Secretary, 28, Finsbury Pavement, E.C. If you are not a nurse, the United Sisters' Friendly Society (secretary for London branch, Miss E. M. Maskell, 7c, Lower Belgrave Street, S.W.) might suit your requirements.

